# GASH: An improved algorithm for maximizing the number of equivalent residues between two protein structures

**DOI:** 10.1186/1471-2105-6-221

**Published:** 2005-09-08

**Authors:** Daron M Standley, Hiroyuki Toh, Haruki Nakamura

**Affiliations:** 1 Institute for Protein Research, Osaka University, 3-2 Yamadaoka, Suita, Osaka 565-0871, Japan; 2 Japan Science and Technology Agency, Institute for Bioinformatics Research and Development (BIRD), Japan; 3 Division of Bioinformatics, Medical Institute of Bioregulation, Kyushu University, 3-1-1, Maidashi, Higashi-ku, Fukuoka, Fukuoka 812-8582, Japan

## Abstract

**Background:**

We introduce *GASH*, a new, publicly accessible program for structural alignment and superposition. Alignments are scored by the Number of Equivalent Residues (NER), a quantitative measure of structural similarity that can be applied to any structural alignment method. Multiple alignments are optimized by conjugate gradient maximization of the NER score within the genetic algorithm framework. Initial alignments are generated by the program Local ASH, and can be supplemented by alignments from any other program.

**Results:**

We compare GASH to DaliLite, CE, and to our earlier program Global ASH on a difficult test set consisting of 3,102 structure pairs, as well as a smaller set derived from the Fischer-Eisenberg set. The extent of alignment crossover, as well as the completeness of the initial set of alignments are examined. The quality of the superpositions is evaluated both by NER and by the number of aligned residues under three different RMSD cutoffs (2,4, and 6Å). In addition to the numerical assessment, the alignments for several biologically related structural pairs are discussed in detail.

**Conclusion:**

Regardless of which criteria is used to judge the superposition accuracy, GASH achieves the best overall performance, followed by DaliLite, Global ASH, and CE. In terms of CPU usage, DaliLite CE and GASH perform similarly for query proteins under 500 residues, but for larger proteins DaliLite is faster than GASH or CE. Both an http interface and a simple object application protocol (SOAP) interface to the GASH program are available at .

## Background

The coordinates of over 30,761 protein structures are currently available at the Protein Data Bank (PDB [1]), and each year thousands of new structures are deposited. A quantitative analysis of this data requires accurate tools for superimposing protein structures, measuring their similarity, and identifying structurally equivalent residues. However, unlike sequence analysis, there is no universally accepted measure of structural similarity. Moreover, even if such a measure existed, structure alignment is so much more complex than sequence alignment, that none the most popular programs available on the Web (e.g., Dali [2,3], CE [4], or VAST [5]) can guarantee an optimal structural alignment in every case. For this reason, it is very useful to have several publicly-available structure alignment tools, as well as a single measure of structural similarity that can be applied to all of them in order to select the best result.

Recently, we introduced an intuitive and convenient measure of structural similarity, the Number of Equivalent Residues (NER), and evaluated several popular structural alignment servers based on this score [6]. By using a single metric (NER) we were able to show that the servers generally converged on the same solution, a result that was not apparent when two metrics (e.g. RMSD and number of aligned residues), or raw scores were used to compare server results.

Another result was that there were occasionally significant differences between servers. This was particularly true for proteins with repeating motifs (e.g. TIM barrels), multiple domains, or in cases where the structurally equivalent residues represent only a small subset of the total. More recently, Levitt and co-workers concluded that there was "wide variation" in alignment quality among different programs and that the performance of any single method was much lower than using the best result from several methods [7]. Our own abservations along these linese motivated us to design a structural alignment algorithm that is robust in locating the global maximum of the NER score even for very difficult cases.

Since the NER score requires an initial alignment or superposition, a straightforward way to add robustness to the optimization algorithm is to increase the number of initial alignments. For this purpose a new alignment program, *Local ASH*, based on the double dynamic programming algorithm, was developed. In contrast to our earlier program, *Global ASH *[8,9], that computed only the globally optimal alignment, the new program computes multiple, locally-optimal alignments.

In addition to accepting multiple initial alignments, the GASH program allows *crossover *between alignments, as is done in genetic algorithms (hence the "G" in GASH). Since both the number of initial alignments and the number of crossovers is an adjustable parameter, the GASH program can make a very good estimate of the true maximum of the NER score for an arbitrary pair of protein structures. One can even import alignments from other programs, and we give an example of combining Local ASH, DaliLite, and CE alignments in this study.

The test-set presented here has been significantly expanded compared to earlier work. In addition to the Fischer-Eisenberg set of structural pairs, our new set consists 3,101 pairs representing many different folds, as defined by SCOP [10]. In addition, GASH is compared directly with the DaliLite and CE executables, allowing CPU time as well as accuracy to be evaluated. As in previous work, accuracy is defined by both the NER score and the number of residues aligned within a given RMSD threshold. In addition, several structure pairs that were not aligned properly by our previous program, Global ASH, are eximined in detail in terms of the alignment of functionally conserved residues.

## Implementation

The overall approach is to globally optimize the NER score in three steps:

1. Produce a set of locally optimal alignments.

2. Parse each alignment into geometrically-consistent sub-alignments using distance matrix comparison.

3. Cross the alignments a fixed number of times and select the best unique set by NER maximization.

**Figure 1:**
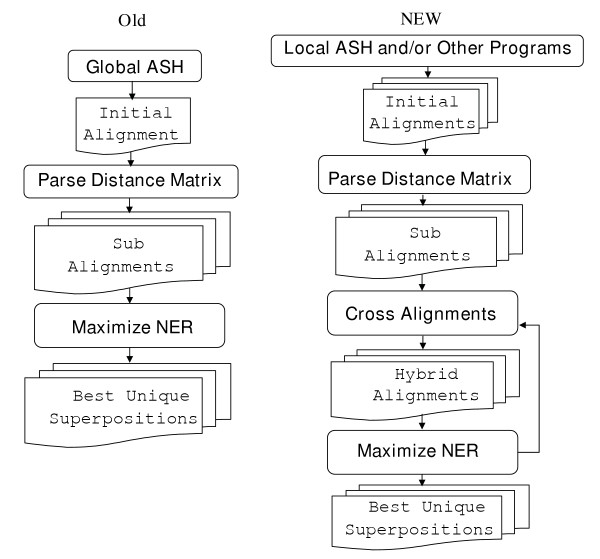
**GASH flowchart**. A flow chart of the Global ASH/NER (OLD) and GASH (New) methods is shown. The key differences between the old and new methods are: the generation of multiple initial alignments, a modified parsing algorithm for generation of sub-alignments, and the further generation of hybrid alignments by crossover.

This procedure as well as our earlier protocol are illustrated in figure 1, and each of the steps is described in more detail below.

### Target function

The NER score has been described in detail previously. In brief, NER is a sum over all aligned residue pairs of a similarity function *S*:



where *k *corresponds to an aligned residue pair, *d*^*k *^is the intermolecular distance between *C*_α _atoms in the aligned pair, and the similarity score is a Gaussian curve with unit amplitude at zero distance:



The parameter *d*_*cut *_defines the tolerance in the similarity score. Since the best value of *d*_*cut *_depends on the problem at hand, we make it an adjustable parameter on our public server. In all calculations a value of 4Å was used.

### Local ASH program

Local ASH is a local structural alignment program that utilizes the double dynamic programming algorithm (DDP) [11]. In DDP, a local frame is used to describe the environment of each residue. A set of vectors from the beta carbon of the residue in question to those of all the other residues in the structure is calculated using the local frame of the residue. The vectors are ordered in the set, according to the position of the destination residue in the primary structure. This set is called the structural environment of the residue. The next step is to form an optimal vector-to-vector correspondence between each pair of structural environments using standard dynamic programming (DP). The similarity between a pair of residues is given by the score resulting from an alignment of the corresponding structural environments. The similarity matrix thus obtained is used in a second DP calculation to yield the residue-to-residue correspondence or alignment. The DP used to evaluate the similarity between a pair of structural environments is called the 'lower level DP', whereas the DP used to solve the residue-to-residue correspondence is called the 'upper level DP'. The method is called DDP, since DP is used at two different levels. Taylor and Orengo further extended the method to local structural alignment [12] by using the Smith-Waterman algorithm for the upper level DP [13].

Both Global and Local ASH use DDP, but with some important modifications. The first modification is a distance cutoff used to define the structural environment. A sphere with a given radius is located at the beta carbon of each residue. The structural environment of a residue is expressed as an ordered set of the vectors from the beta carbon of the residue to those of the residues whose beta carbons are within the sphere. The modified environment is called the local environment. The similarity between a pair of residues is calculated as the alignment score between the corresponding local environments. The similarity obtained from the comparison of the local environments is used for the local structural alignment. In order to avoid confusion with the distance cut-off used in the NER score, discussed below, we will refer to the ASH distance cutoff as the *alignment radius*.

Local ASH uses the Smith-Waterman algorithm for local structural alignment. When a pair of structures share multiple structural similarities that can not be expressed in a single alignment, the trace-back procedure is repeated in order to enumerate high-scoring solutions. After each trace-back operation, the scores corresponding to the alignment path and the neighboring region in the similarity matrix for the upper level DP are cleared. This ensures that each alignment will trace out a unique path. Orengo and Taylor introduced a window surrounding the local alignment path as the region to be cleared [12]. In contrast, Local ASH adopts the declump algorithm, which has been developed for local sequence alignment [14]. Local ASH also has the option to detect local similarity derived from circular permutation [15], although the option was not used in this study.

For results described here, the alignment radius we set to 14Å, and the maximum number of alignments output was 25. The internal gap opening and extension penalties for the lower level DP calculation were set to 10 and 0.5, respectively. For terminal regions the gap penalties were all zero. For the upper DP calculation, the gap opening and extension penalties were also set to 10 and 0.5, respectively. The Local ASH source code can be freely downloaded [16].

### Parsing alignments using distance matrix comparison

Local ASH can not "see" beyond the alignment radius. If two or more regions of structural similarity exist and are separated by a distance greater than the alignment radius, an alignment may be constructed that runs through both regions. If these multiple regions do not correspond to a superposition with the same rotation and translation values, there will be more than one maximum in the landscape of the NER scoring function for the alignment. The distance matrix comparison step is used to decouple such geometrically-distinct sub-alignments.

A necessary condition for an alignment to be geometrically consistent is for all of the intramolecular distances between aligned residues in one structure to be approximately the same length as the corresponding distances in the other structure. Such a comparison of distance matrices forms the basis of the Dali target function [2]. Here we use it as a constraint. The sub-alignments thus constrained correspond to sets of residue pairs where the difference in corresponding intramolecular distances agree within a specified tolerance.

The parsing is iterative: the first aligned pair of residues (*i*_*q*_,*i*_*t*_) initiates a sub-alignment (Here, the subscript *q *refers to the query and *t *to the template). Then we attempt to add a second aligned pair (*j*_*q*_,*j*_*t*_) to this sub-alignment. If we find that any of the intramolecular distances in the query  differ by more than a cutoff value from the corresponding distance in the template, , the residue pair (*j*_*q*_,*j*_*t*_) is rejected from the sub-alignment; otherwise, it is accepted. If the pair is rejected from all existing sub-alignments, a new sub-alignment is initialized. Each subsequent pair of residues is compared to each existing sub-alignment in this manner until all residue pairs in the original alignment have been accounted for.

There are two differences between the algorithm used in GASH and that used by us previously: first, a residue pair can be in more than one sub-alignment, as long as the difference cutoff is satisfied. Second, the cutoff used in the current work is 10Å rather than 20Å.

**Figure 2:**
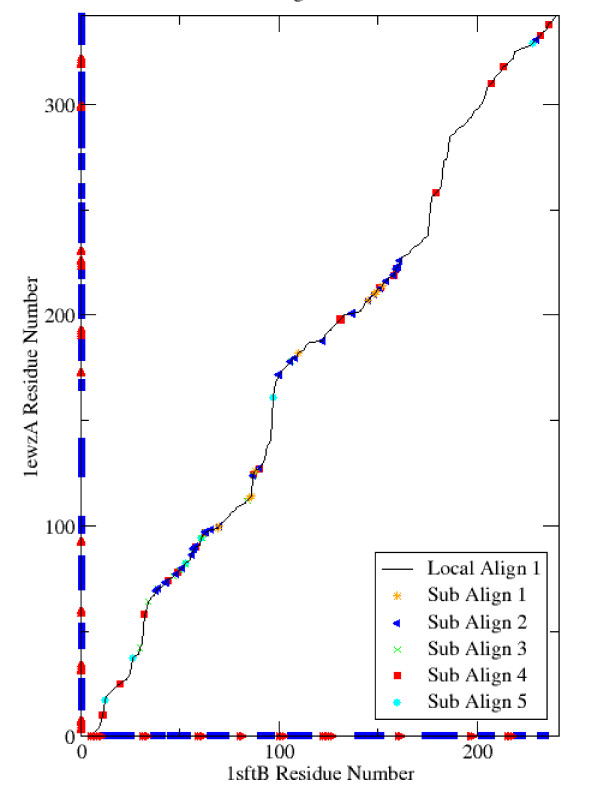
**Alignment parsed by distance matrix**. The parsing of a single local alignment into geometrically consistent sub alignments is illustrated. Only five sub-alignments are shown, and consecutive aligned residue pairs belonging to the same sub-alignment are represented by a single point in order to make the plot easier to see. The secondary structure (helices in blue and strands in red) is plotted along the axis.

In figure 2 we show some of the sub-alignments from a single local alignment between 1sftB and 1ezwA. Although about two-thirds of the aligned pairs have not been plotted, in order to make it easier to see individual pairs, it can be seen that the aligned pairs from a particular sub-alignment are not clustered together in sequence number but are distributed over a wide range of values.

### NER maximization

NER maximization involves first optimizing the superposition, given an initial alignment, and then re-optimizing the alignment based on the new superposition. For optimizing the superposition, we first minimize the *C*_α _RMSD of the aligned residues, then directly maximize the NER score by conjugate gradient optimization. The method of Mclachlan is used for RMSD minimization [17]. For the conjugate gradient optimization step we use the Fletcher-Reeves-Polak-Rebiere method as implemented in the Numerical Recipes program frprmn [18].

We then re-calculate the alignment based on the residue-based similarity score (eqn. 2) using an ordinary dynamic programming calculation [19]. This re-alignment step is similar in approach to that used by May and Johnson [20,21] as well as Gerstein and Levitt [22]; however, in our case we do not iterate between superposition and alignment. From the new alignment, we calculate the optimal NER_4 _score. Note that the final NER score will be sensitive to the relative gap penalty used in the dynamic programming step, so one must be sure to use the same parameters when comparing alignments. The best choice for the gap penalty depends on the problem at hand. For all results presented here, we use .25 for internal gap opening, and .125 for internal extension.

### Crossing alignments

Genetic algorithms have been used to generate alignments [23] or superpositions [20,21]. Here we use Local ASH to generate a very reasonable set of initial alignments, and use the crossover operation to exchange information between this initial set of alignments in order to obtain a globally optimal solution. We do not require the mutate, or other local operations, as we are not attempting to generate new information at this point, and because the NER maximization procedure can locally optimize an imperfect alignment generated by the crossover step. Crossover is the only stage where a random element is explicitly introduced into the procedure, and thus, in combination with the choice of initial alignments, is a point where the extent of sampling can be adjusted. The crossover algorithm used in GASH is as follows:

1. A stack to hold hybrid alignments is initialized with a fixed maximum length.

2. Two alignments as well as a splice-point in one of them are chosen at random.

3. The splice point in the second alignment is chosen so that the C-terminal portion is as long as possible without containing any of the residues in the N-terminal portion of the first alignment.

4. The alignments are crossed with a single progeny: the N terminal portion of alignment 1 and the C-terminal portion of alignment 2.

5. The CA RMSD of the new alignment is minimized, and the NER score calculated from the RMSD-minimized superposition. If the stack-size has not reached the maximum value, the new alignment is saved; otherwise, if this new NER score is greater than the lowest saved NER score in the stack, the lowest saved alignment is replaced by the new alignment. (This means we don't have to sort the stack but just keep track of the worst saved solution.)

6. Return to step 1 a fixed number of times (*N*_*try*_).

The entire cross-over cycle is repeated *N*_*start *_times, starting from the same initial set of alignments. After each cycle, the top *N*_*top *_alignments are subjected to full NER maximization. The values *N*_*start*_, *N*_*try*_, and *N*_*top *_are all parameters that can be used to adjust the extent of sampling, as described in the GASH Variants section, below.

**Figure 3:**
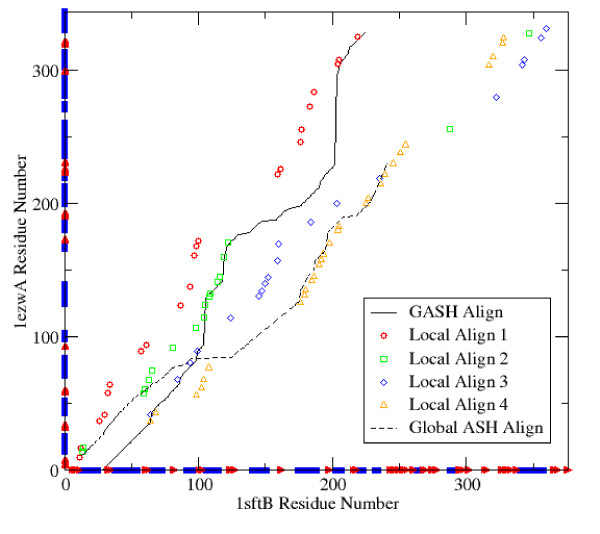
**Local and global alignments**. The crossover operation is illustrated here by showing the final GASH alignment between 1sftB and 1ezwA. Four of the initial Local ASH alignments are shown as scatter plots, which are partially sampled by the final GASH alignment, as well as the Global ASH alignment.

In figure 3 we show the final GASH alignment between 1sftB and 1ezwA along with some of the initial alignments produced by Local ASH. We can see in this example that the final GASH alignment samples at least three different local alignments, and that this solution is completely different from the one obtained by Global ASH.

### Saving unique results

It should be emphasized that, while in the present study we focus on global optimization, the GASH program produces multiple solutions. The selection of final solutions involves sorting the saved results and removing lower-scoring alignments from the list that are similar (as defined by an RMSD threshold) to higher-scoring ones. On the GASH server, the RMSD threshold, as well as an NER threshold for accepted solutions, can be adjusted in order to modify the number of retained alternate solutions.

### DaliLite program

The DaliLite program was obtained from the FSSP [24] server [25]. The DaliLite NER scores were calculated by dynamic programming as described above using the DaliLite superposition without modification. The calculation was identical to that used to evaluate the superposition of the GASH structures.

### CE program

The CE program was obtained from the CE server [26]. The NER scores were calculated in a manner identical to that used for Dali, except that the superimposed coordinates were generated by our own script based on the rotation matrix and translation vector produced by the CE program.

### GASH variants

In order to assess the necessity and sufficiency of the crossover step, we report results using four versions of GASH: Default, No Cross-over, High Cross-over, and Meta. These variants are summarized below.

#### Default GASH

The default settings for *N*_*start*_, *N*_*try*_, and *N*_*top *_are 2,100, and 20, respectively. Initial alignments are generated by Local ASH.

#### No cross-over GASH

In order to assess whether the extent of crossover specified by the default parameters is necessary, GASH was run with no crossing over (*N*_*start *_= 0).

#### High cross-over GASH

In order to assess whether the extent of crossover specified by the default parameters is sufficient, GASH was run with extensive crossing-over (*N*_*start *_= 200).

#### Meta GASH

In order to assess whether the initial set of alignments generated by Local ASH is sufficient, alignments extracted from DaliLite and CE were added to the initial set. In the case of DaliLite, we used all alignments generated by the program as well as those extracted from the DaliLite superpositions, using the re-alignment procedure, described above.

### Test sets

Two test sets are used to assess the performance of GASH. The first was derived using SCOP[10] and FSSP[24]. The second was derived by Fischer and Eisenberg has been used by others[4] to benchmark structure comparison algorithms. We will refer the these as the SCOP-FSSP and the Fischer-Eisenberg sets, respectively.

The SCOP-FSSP set was generated by a two-step procedure. First, SCOP [10] entries from different families were chosen by hand then checked to see if they were among the pre-computed lists of structure pairs at the FSSP [24] server. One entry, trehalose-6-phosphate synthase, was not found on FSSP, so trehalose-6-phosphate phosphatase related protein (1u02A) was used instead. The resulting 17 structures are referred to as "queries". Next, for each query, all structural neighbors were taken from the FSSP server. These structures are referred to as "templates". The template list was obtained by browsing the "FOLD Index," based on the PDB90 representative set of proteins [27], starting from the PDB ID of the SCOP entry. Note that while no two templates share more than 90% sequence identity, one of the templates is likely to have a high sequence similarity to the query as this closest matching representative is used to select the best pre-computed list. Examples are 1sftB (query) 1bd0A (representative template) and 1ab8A (query) 1cs4B (representative template).

A number of multi-domain queries were selected in this way under the assumption that these structures would yield a diverse and challenging set of templates. The result was a set of 3,593 structure pairs containing many different types of proteins. Since FSSP, which uses Dali to generate structure pairs, was used to create our test-set, there should be no bias toward GASH in the SCOP-FSSP set. Subsequently 491 of these structure pairs were eliminated because one or more of the programs failed to produce a meaningful alignment (see below), resulting in a set of 3,102 structure pairs that were used for the present analysis. The set of queries, along with their fold classifications is given in Table 1.

**Table 1:** Query List. The set of queries used to generate the SCOP-FSSP set is shown. The chain ID, if non-blank, is appended to the PDB ID (column 1). The number of residues refers to the entire protein chain. The Class and Fold are taken from SCOP, except in the case of 1u02A, which was not classified by SCOP.

**PDB ID**	**Protein Name**	**Nres**	**Class**	**Fold**
1ab8A	Type II Adenylyl Cyclase C2 Domain	208	α+β	Ferredoxin-like
1bxrA	Carbamoyl Phosphate Synthetase(CPS)	1104	1. α 2. α/β 3. α/β 4. α/β 5. α/β 6. α+β	1. CPS connection domain 2. Swivelling β/β/α domain 3. Flavodoxin-like 4. Methylglyoxal-like 5. PreATP Grasp domain 6. ATP Grasp
				
1frvA	Nickel-Iron Hydrogenase (NIH)	293	1. α+β 2. α+β	1. NIH Large subunit 2. NIH Small Subunit
				
1mniA	Myoglobin	184	*α*	Globin-like
1qgtB	Hepatitis B Viral Capsid	174	*α*	Hepatitis B Viral Capsid
1u02A	Trehalose-6-Phosphate Phosphatase Related Protein	253	*α*/β	
				
1bgw	DNA topoisomerase II, C-terminal fragment (residues 410–1202)	709	Multi-dom α+β	Type II DNA Topoisomerase
				
1dwuA	Ribosomal Protein L1	244	Multi-dom α+β	Ribosomal Protein L1
1gqeA	Polypeptide release factor 2	385	Multi-dom α+β	Polypeptide release factor 2
1nvbB	Dehydroquinate Synthase (DHQS)	422	Multi-dom α+β	DHQS-like
1r6fA	Low Calcium Response Protein V (LcrV)	303	Multi-dom α+β	Virulence-associated V antigen
1udyA	Medium Chain acyl-CoA Dehydrogenase	416	1. α 2. Multi-dom α+β	1. Bromodomain-like 2. Acyl-CoA dehydrogenase NM domain-like
				
1bwwA	Immunoglobulin Light Chain Kappa Variable Domain	140	*β*	Immunoglobulin-like β-sandwich
				
1e03L	Antithrombin	454	Multi-dom α+β	Serpins
				
1kyqB	Bifunctional dehydrogenase/ferrochelatase Met8p	298	1. α/β 2. Multi-dom α+β	1. NAD(P)-binding Rossmann-fold domains 2. Siroheme Synthase Middle domain-like
				
1obaA	Endolysin	369	1. β 2. α/β	1. β-hairpin stack 2. TIM β/α-barrel
				
1sftB	Alanine Racimase	411	1. β 2. α/β	1. Domain of α+β subunits of F1 ATP synthase-like 2. TIM β/α-barrel

The Fischer-Eisenberg set was taken from table VI in Shindyalov and Bourne [4].

## Results

### Overview

The results were generated by running each program on the command line on one of 14 identical personal computers (Intel Pentium4 processor, Linux RedHat 8.0 operating system). In order to make a fair comparison, the same re-alignment procedure was used to evaluate the NER scores and the numbers of aligned residues for all alignment methods. The numbers of aligned residues were computed for three RMSD cut-offs: 2,4, and 6Å, which we will refer to as N2, N4, and N6, respectively. Occasionally one or more of the programs failed to produce a result, or only aligned a few residues. Since we could not distinguish between software errors (e.g. parsing the initial PDB file or some incompatibility between our system and one of the programs) and true algorithmic deficiencies, we eliminated any template from the list if any one of the methods failed to produce an NER score greater than 10. Based on the higher number of failures for DaliLite, CE and Global ASH (171,180, and 100, respectively) compared to the default, no crossover, and high crossover GASH (58,64, and 56, respectively) it is unlikely that eliminating templates in this way biased the results in favor of GASH.

**Figure 7:**
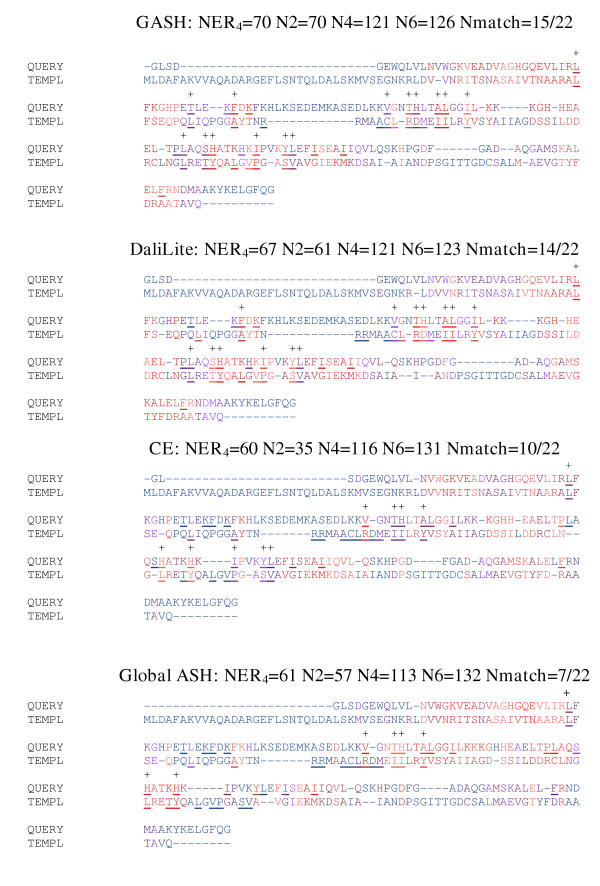
**Myoglobin aligned to Phycocyanobilin. **Myoglobin (1mniA, query) aligned to Phycocyanin (1phnB, template). Residues that bind heme in 1mniA and phycocyanobilin in 1phnB are underlined, with matches indicated by a + and the total number of matches reported at the top of each alignment. The color scale used in this figure is identical to that of figure 6. The secondary structure assignments, residue equivalences, and terminal gaps have all been omitted in order to save space.

**Figure 8:**
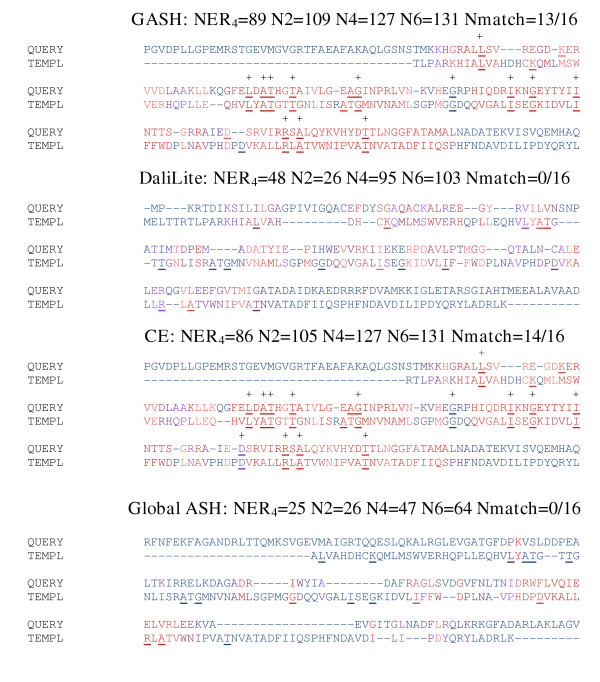
**Carbamoyl phosphate synthetase aligned to methylglyoxal synthase. **Carbamoyl phosphate synthetase (1bxrA, query) aligned to methylglyoxal synthase (1egh, template). Conserved residues in the methylglyoxal synthase-like superfamily are underlined, with matches indicated by a + and the total number of matches reported at the top of each alignment. The format used in this figure is identical to that of figure 7.

**Figure 9:**
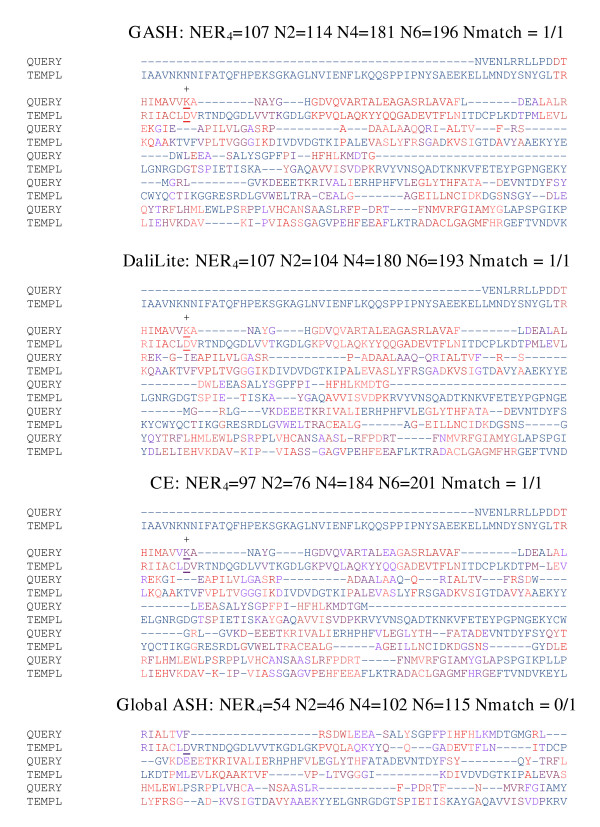
**Alanine Racimase aligned to imidazole glycerol phosphate synthase. **Alanine Racimase (1sftB, query) aligned to imidazole glycerol Phosphate synthase (1jvnA, template). A pair of function residues found the TIM barrel are underlined, with matches indicated by a + and the total number of matches reported at the top of each alignment. The format used in this figure is identical to that of figure 7.

**Figure 10:**
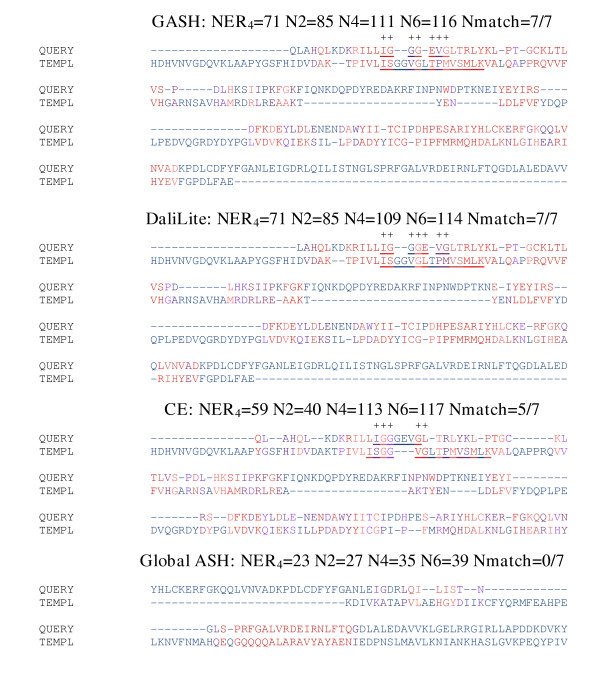
**Met8p aligned to flavohemoglobin. **Met8p (1kyqB, query) aligned to Flavohemoglobin (1cqxA, template). The NAP(p)-binding loop residues are underlined, with matches indicated by a + and the total number of matches reported at the top of each alignment. The format used in this figure is identical to that of figure 7.

**Figure 11:**
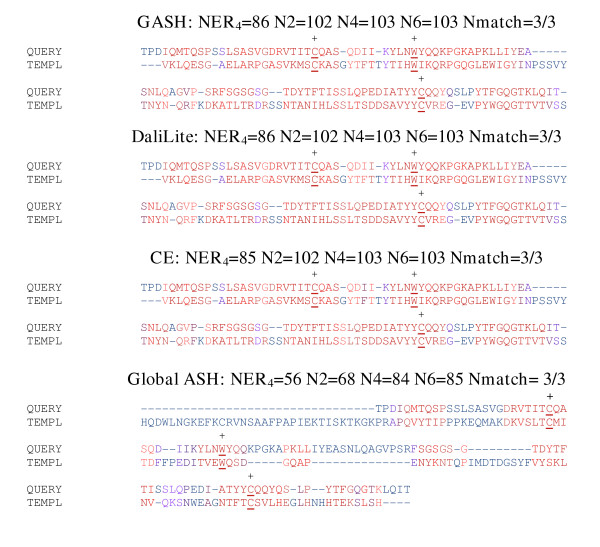
**Immunoglobulin Light Chain Kappa Variable Domain aligned to antibody for phenobarbital**. Immunoglobulin Light Chain Kappa Variable Domain (1bwwA, query) aligned to antibody for phenobarbital (1igyB, template). The characteristic disulfide bond and Thr residues are underlined, with matches indicated by a + and the total number of matches reported at the top of each alignment. The format used in this figure is identical to that of figure 7.

Averages of all similarity measures as well as the number of internal gaps were computed for each query set as well as for the entire set of results (see Additional file 1). In addition, the average CPU time per alignment is given for each query set and for the entire set of results in table 2. From the SCOP-FSSP set, a sub-set of 5 structure pairs that were not aligned properly by Global ASH are discussed in detail and shown in figures 7, 8, 9, 10, 11. Finally, we present results for the smaller Fischer-Eisenberg data set (see Additional file 2).

**Table 2:** Timings. The average CPU times for each query from the SCOP-FSSP set using each of the 6 programs is shown. The Meta Gash program CPU can be closely approximated by summing the GASH default, DaliLite, and CE columns.

**Query**		**Average CPU (seconds)**					
		**GASH**			**Global**	**GASH**	**GASH**
**ID**	**Nres**	**Default**	**Dalilite**	**CE**	**ASH**	**No Cross**	**High Cross**

1bwwA	110	4	4	13	4	3	8
1qgtB	144	7	6	17	10	6	10
1mniA	154	4	4	13	5	4	9
1ab8A	178	5	3	15	6	5	9
1dwuA	214	13	9	18	17	13	20
1u02A	223	13	10	19	15	13	18
1frvA	263	14	10	18	16	13	20
1kyqB	268	15	10	20	20	15	21
1r6fA	273	7	5	17	10	7	11
1obaA	339	27	23	24	31	25	34
1gqeA	355	15	10	22	19	14	20
1sftB	381	25	22	24	28	24	32
1udyA	386	14	12	24	20	13	19
1nvbB	392	25	16	23	30	25	32
1e03L	424	22	22	18	31	21	36
1bgw	680	40	25	45	53	39	48
1bxrA	1074	60	28	61	83	58	69

### Output files

**Figure 4:**
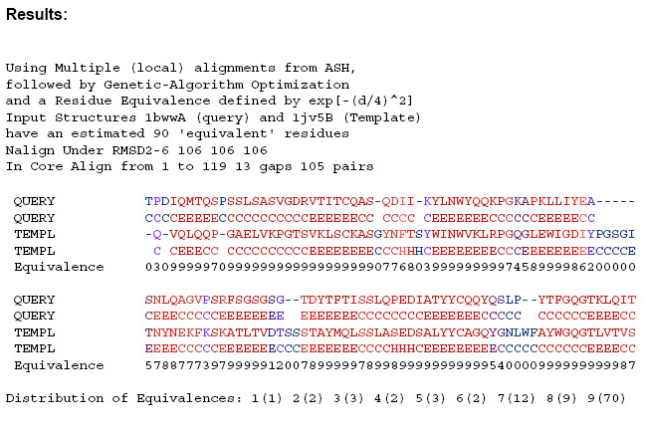
**GASH alignment format. **The alignment between 1bwwA and 1jv5B using default GASH is shown. In addition to the total NER score (eqn. 1), the residue-based similarity score (eqn. 2) was evaluated and scaled to integer values between 0 and 9. The distribution of such equivalences is reported at the bottom of the alignment. In order to roughly define the beginning and end of the most important parts of each alignment the first and last set of 5 continuous residues where the average similarity score was 5 or more was located. We refer to this region as the core alignment, and report the number of gaps and aligned residue pairs within the region. Also, the number of residues aligned under the three RMSD cutoffs, N2-6 are indicated. The alignments were written out with the residue pairs and secondary structure color coded by the similarity scale (with red the most and blue the least similar), making it easy to recognize regions of structural similarity.

The raw data resulting from each of the 3,102 structure pairs using 7 different methods is too large to be presented in tabular form here. For this reason, we have prepared a link to the data on our server [28]. The main page summarizes the results for each query and is reproduced in text form in Additional file 1. The HTML table contains links for each query containing individual scores for each structure pair using each of the 7 alignment methods. Within each query page there are links to each alignment and superposition (in PDB format). An example of the alignment between 1bwwA and 1jv5B is given in figure 4.

### Correlation between NER score and the number of aligned residues

Although the main focus of this work is on optimization, we first consider whether the NER score is a valid target function for structural alignment by comparing it to a more familiar metric: the number of aligned residues under a given RMSD.

**Figure 5:**
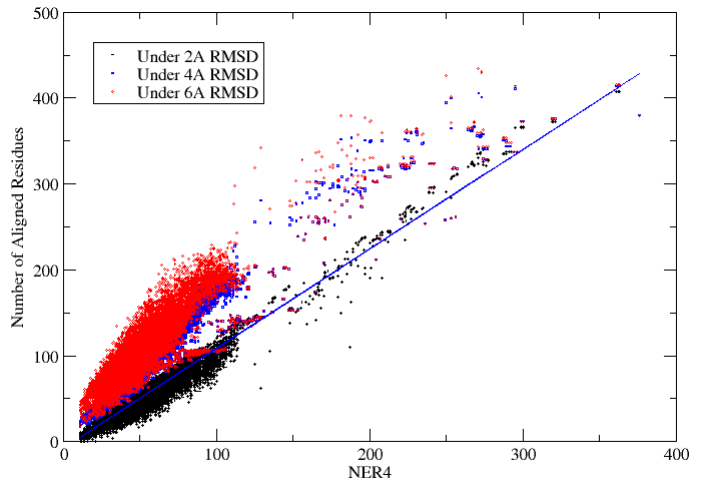
**Number of aligned residues under a given RMSD. **The correlation between NER_4 _and the number of aligned residues under three cut-offs is shown. The entire set of alignments from 3,102 structure pairs and 7 alignment methods was used to make this plot. The slope between NER_4 _and the number of aligned residues under 2Å was 1.2 with a correlation coefficient of .97.

In figure 5 we plot NER4 versus N2, N4, and N6 for all 3,102 structure pairs in the SCOP-FSSP data set using all 7 alignment methods. Since RMSD is an average over a set and NER is a direct sum of normalized values, we do not expect to see an exact agreement; nevertheless, over a broad range of values, the correlation between the different numerical measures is approximately linear. The slope is closest to unity when the smallest RMSD cutoff (2Å) is used (slope 1.2, correlation coefficient .97).

The fact that NER is a direct sum makes it much easier to optimize than the number of aligned residues under a given RMSD cutoff. (For example, it can be maximized directly by dynamic programming or by conjugate gradient optimization.) This fact coupled with the nearly linear agreement between the two measures validates the utility of NER as a target function for structural similarity.

### Optimization performance

First we consider the improvement of GASH compared to Global ASH from the summaries given in Additional files 1 and 2. In terms of optimization of the NER score (or any of the other measures), GASH consistently out-performs Global ASH. An improvement of approximately 10 residues is seen in every query average, with the exception of 1e03L, where the two programs agreed on average. The improvement in terms of alignment accuracy is achieved while at the same time decreasing the CPU time per alignment (table 2).

Next we consider the performance compared to DaliLite and CE. GASH consistently aligned an equal or greater number of residues than either DaliLite or CE, independent of the measure used to define accuracy. The improvement relative to CE is particularly dramatic, with an average of 7–19 more residues aligned (depending on the measure used). With DaliLite, the improvement (4–8 residues, on average) was not as dramatic, but it was consistent across most query sets. In terms of CPU usage, CE was the slowest for proteins in the 100–273 residue range, and DaliLite was several seconds faster than GASH in this size range. In the 339–424 residue range, CPU times differ between the four programs by only a few seconds, on average. The greatest difference in CPU usage is seen for the largest structures: DaliLite uses only 25 and 28 seconds, for 1bgw (680 residues) and 1bxrA (1074 residues), but both GASH and CE run for longer times(approximately 40 and 60 seconds, respectively).

It must be emphasized that these results represent use of the DaliLite and CE programs as is, without any changes in the source code for this difficult set of alignment problems. In the hands of the authors, CE or DaliLite might well yield higher NER scores and/or lower CPU times.

### Sufficiency of Local ASH initial alignments

We address the question of coverage in the initial set of alignments by comparing the default GASH performance with Meta GASH, where alignments from DaliLite and CE were added to the initial set. Since GASH uses only the crossover operation from the genetic algorithm, not mutation, the only new information it can generate is in the re-alignment step. DaliLite and CE both generate alignments by completely different algorithms from each other and from Local ASH, so if there is not enough information in the initial set of Local GASH alignments, we should see an improvement in the accuracy when alignments from DaliLite and CE are added.

In terms of the NER score, there is a slight average improvement of 2 aligned residues upon using Meta GASH. This improvement can be observed consistently across most query sets, showing that we are not always locating the exact global optimum in the NER score when using default GASH. However, these differences are not great enough to justify using Meta GASH routinely, since the CPU usage would be approximately 3 times that of default GASH.

### Necessity and sufficiency of crossover

**Figure 6:**
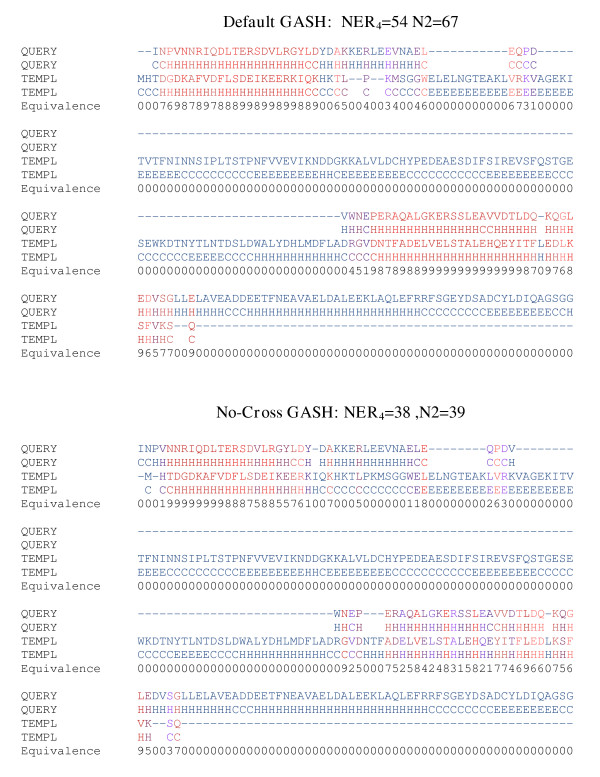
**Default GASH vs. no crossover. **The default GASH protocol is compared to GASH without crossover for 1gqeA (query) aligned to 1p32A (template). The NER equivalence (eqn. 2) is indicated numerically, on a 0–9 scale, and by color (with red the most and blue the least similar).

In order to determine if the crossover is both necessary and sufficient, we compare default GASH to GASH without crossover and to GASH with high crossover. The no-crossover results are not as good as those of default GASH, but the improvement is not very dramatic, on average. In fact, no-crossover GASH is slightly better on average than DaliLite, and at a competitive CPU usage. However, if we consider particular cases, such as the alignment between 1gqeA and 1p32A (figure 6), the difference in NER4 is 16, and in the number aligned under an RMSD of 2Å is 28 residues. Such particular cases, as well as the fact that the difference in CPU between default and no-cross GASH is only a few seconds at most, justifies the use of the crossover operation. In contrast, when we increase the crossover by a factor of 100 we do not see an improvement in any of the similarity measures, on average. This strongly suggests that the extent of crossover in the default program is both necessary and sufficient.

### Specific examples from SCOP-FSSP set

Here we examine the quality of 5 pair-wise alignments in detail. In addition to numerical measures, such as the number of equivalences, we consider functional information, where available. The examples chosen represent cases where GASH outperformed Global ASH in terms of the NER score, and include all-α, all-β, and mixed α/β folds. In all examples, "GASH" refers to the default GASH method. All sequence identities quoted are obtained by running the entire sequence for both query and template at the University of Southampton SBDS server [29], which uses Lipman and Pearson's algorithm [30].

#### Myoglobin (1mniA) aligned to phycocyanin (1phnB)

Myoglobin and phycocyanin belong to the same SCOP fold group but have different functions. Alignment of the two sequences yields an identity of 15%. Myoglobin utilizes a heme group to transport oxygen and phycocyanin binds a phycocyanobilin chromophore for light harvesting. The heme group bound by myoglobin and the chromophore bound by phycocyanin are positioned similarly [31]. Although 1mni is a double-mutant form (with two of the binding-site residues switched), the mainchain RMSD from the native is only .25Å [32]. Thus we expect that in the proper structural superposition, the residues responsible for binding the prosthetic groups would be aligned. Figure 7 shows the GASH, DaliLite, CE, and Global ASH alignments between 1mniA and 1phnB. In terms of NER4, GASH (70) and DaliLite (67) perform similarly well, while CE (60) and Global ASH (61) perform similarly poorly. The N2 -4 scores are more varied, but follow the same general trend. There are 22 heme/chromophore binding residues in each structure. Their distribution in sequence is such that a perfect match seems unlikely; nevertheless, the number of matches correlates with the NER score except in the cases with low matches: CE aligns 10 of the functional residues, whereas Global ASH only aligns 7. In the case of DaliLite (14) and GASH (15) we can see that there are only slight differences in the alignment, and that the one pair of functional residues aligned by GASH but not by DaliLite is a borderline case: they are nearly aligned in the DaliLite alignment, and in fact, occur at a point of fairly poor structural superposition in both alignments.

#### Carbamoyl phosphate synthetase (1bxrA) aligned to methylglyoxal synthase (1egh)

The C-terminal domain of 1bxrA and the entire structure of 1egh both belong to the methylglyoxal synthase fold [10,33-35]. However, the shapes of their active sites, as well as the functions of the two proteins differ significantly. As a result, we cannot use the same approach used to check the 1mniA-1phnB alignments to check the 1bxrA-1egh alignments. Fortunately, even though the overall sequence identity is only 6%, these two proteins contain conserved residues. The set of conserved residues was defined in the following way: each sequence was used as a query to the Conserved Domain Database[36], yielding an alignment to the consensus sequence of the methylglyoxal synthase-like domain; the 1bxrA-1egh alignment was then constructed by aligning the consensus sequences of from each alignment. Conserved residues were defined to be those residues that were aligned and identical to the consensus sequence. This small set of conserved residues are distributed throughout the domain. As figure 8 shows, GASH aligned all of the conserved residues correctly, with two exceptions: The first conserved Lys residue does not superimpose structurally; also, Asp 1025 in 1bxrA should be aligned to Asp 101 in 1egh; however, it is aligned instead to residue 99. Coincidentally, residue 99 happens to be an aspartic acid as well, but this appears to be an accident – the conserved Asp is residue 101. The real problem here lies in the fact that GASH considers only Cα residues in constructing the equivalences used to compute the final alignment. In fact, the side-chains in residues 1025 and 101 are much closer than those of 1025 and 99. In other words, the superposition is fine, but the alignment computed from the superposition is less than optimal, due to the exclusive use of Cα residues in the scoring function. The DaliLite alignment completely misses the C-terminal domain in 1bxrA, and instead aligns 1egh to the N-terminal pre ATP grasp domain. CE produces an alignment that is almost identical to GASH. In terms of the numerical measures, the GASH alignment is slightly better, although CE aligns all of the conserved residues correctly, with the exception of the first Lys, including the aforementioned Asp. Local ASH incorrectly aligns 1egh to the connection domain in 1bxrA.

#### Alanine racimase (1sftB) aligned to imidazole glycerol phosphate synthase (1jvnA)

Alanine Racimace and imidazole glycerol phosphate synthase share a TIM barrel domain with the active site located at the top of the barrel [10,37-39]. There is only 17.5% sequence identity between the two, and they do not share a common ligand, so we can not find obvious markers as we did in the previous examples. However, both proteins are involved in peptide biosynthesis, and each contains one catalytic residue in the TIM domain: Lys 19 in 1sftB acts as a proton acceptor specifically for D Alanine and Asp 245 in 1jvnA makes hydrogen bonds with imidazole glycerol phosphate, a precursor in the histidine synthetic pathway. As figure 9 shows, GASH and DaliLite yield essentially the same alignment, and align the Lys-Asp pair. Although there is no reason to assume a priori that the functional residue in the two proteins should align, the fact that it does is probably not an accident. The CE alignment is somewhat lower in quality by numerical measures, but aligns the functional residue pair as well. The Global ASH alignment is completely different from the rest, and much lower in quality. In our previous study of Global ASH we also found that correctly pairing the beta strands in TIM barrel structures was non-trivial, due to the 8-fold symmetry of the barrel [6].

#### Met8p (1kyqB) aligned to flavohemoglobin (1cqxA)

Both 1kyqB and 1jvnA contain a NAD(p)-binding Rossmann domain [31,40], but the sequence identity is low (15.5%), and there are significant differences in the binding pocket as well as the topology of the fold. 1kyqB contains an extra anti-parallel strand at the edge of the sheet. Interestingly, both structures contain a long, extended, and highly charged loop; however, although the loop occupies a similar spatial position in each molecule, the location of this loop in the primary structure is different. It is difficult to find many functional or conserved residues that are paired in the alignment. The NAD binding loop is much longer in 1cqxA than 1kyqB, so we see a distortion in the alignment precisely at this point. However, the general position of the NAD(p)-binding residues can be used to assess the alignments. In terms of the numerical measures, GASH and DaliLite perform similarly, and both align the loop interacting with NAD(p) as well as can be expected. By any measure the CE alignment is not as accurate, and the Global ASH alignment is a failure, aligning a small fragment from two completely different domains (figure 10).

#### Immunoglobulin light chain kappa variable Domain (1bwwA) aligned to antibody for phenobarbital (1igyB)

Both 1bwwA and 1igyA are immunoglobulins, and contain the characteristic β-sandwich fold. 1bwwA is a single-domain structure, but 1igyB is an intact monoclonal antibody and contains both a variable domain and three constant domains[41]. Thus, the problem of aligning these two structures consists of identifying the best structural match among 4 domain choices. All of the domains in these two structures have the characteristic disulfide bridge and a Trp group located near the bridge. From the standpoint of aligning the correct domain, aligning the Cys and Trp residues, and from the numerical scores, GASH, DaliLite, and CE all succeed and find the exact same solution; Global ASH, on the other hand aligns 1bwwA to one of the constant domains and gets much lower numerical scores. On the other hand, Global ASH does get the functionally conserved residues from the constant domain aligned correctly to those in the variable domain (figure 11).

### Fischer-Eisenberg set

In Additional file 2 we summarize 10 structure pairs from the Fischer Eisenberg data set. The structures in this set are generally smaller than those in the SCOP-FSSP data set, so it is not surprising that the differences between methods are not as large. The general trend, in terms of both numbers of aligned residues and NER score is the same as in the SCOP-FSSP results: Default GASH ≥ DaliLite ≥ CE, but the differences are probably not significant. This suggests that the differences between methods only becomes important when there are multiple domains and/or multiple regions of structural similarity.

## Conclusion

The primary goal of this study was to design an algorithm that reliably maximizes the NER score for an arbitrary pair of protein structures. The results here indicate that the GASH program is successful in this regard, and that the extent of sampling can easily be increased, if necessary, by adding more initial alignments. Although we have not yet optimized every parameter used in GASH, the results in terms of NER and other scores using default parameters is encouraging. From looking at the dependence on crossover and on the initial alignment set, we can surmise that most of the improvement relative to Global ASH is due to the use of multiple Local ASH alignments.

The Dali algorithm has recently been validated extensively against CATH classifications using receiver operating characteristics in two studies [7,42], however in one of these studies [7], the quality of Dali alignments was found to be inferior to CE. Based on our smaller study Dali alignments appear to be more accurate than CE alignments, by any measure. Perhaps eliminating structure pairs from the test set that could not be aligned by one or more of the programs had some effect on the results. Since CE failed in this regard slightly more often than DaliLite, however, it seems unlikely that this had any effect. GASH has not yet been benchmarked on such a comprehensive test set or against fold classifications, such as CATH or SCOP.

Even with a test set of 3,102 structure pairs, the improvement of GASH relative to Global ASH, both in terms of accuracy and CPU usage, is unambiguous. Moreover, as the specific examples illustrate, a higher NER score correlates well with "correctness" in terms of matching important residue pairs. The one case where GASH misaligns a residue pair found by CE immediately suggests an obvious improvement to the program: using side-chain atoms in addition to Cα atoms to define the equivalence. We intend to incorporate this improvement, as well as to look at a more comprehensive set of structure pairs in the near future.

## Availability and requirements

In addition to web access through both a CGI and java interface, we have developed a Simple Object Access Protocol (SOAP) server that allows GASH to be run remotely on the command-line. Sample java and Perl client programs are available for accessing the SOAP server. The java programs require installation of the Apache Axis library [43] and the Perl programs require installation of the SOAP-Lite perl module. All three interfaces are described at the GASH server .

## Supplementary Material

Additional File 1 **Summary of SCOP-FSSP set results**. For each query, the number of residues, number of structural templates (Ntmp), and results from each of the 7 alignment methods, are shown. For each alignment method, the NER_4 _score (NER), number of gaps in the core alignment (Gp), and number of aligned residues below three RMSD cutoffs (N2,N4,N6) are reported. Each entry in the table represents an average over the number of templates specified in column 3. The averages on the last row refer to the total set of 3,102 structures. In the case of the number of templates, the sum, rather than the average, is given. Click here for file

Additional File 2 **Summary of Fischer-Eisenberg set results**. For each structure pair, the PDB ID and number of residues, as well as results from each of the 7 alignment methods, are shown. For each alignment method, the NER_4 _score (NER), number of gaps in the core alignment (Gp), and number of aligned residues below three RMSD cutoffs (N2,N4,N6) are reported. In the last row, the averages over all ten structure pairs are given. Click here for file
