# Supplementary material for: GASH: An improved algorithm for maximizing the number of equivalent residues between two protein structures
Source: BMC Bioinformatics. 2005 Sep 8;6:221. doi: 10.1186/1471-2105-6-221 (PMC1239909; doi:10.1186/1471-2105-6-221)
Supplement: Additional File 1 — Summary of SCOP-FSSP set results. For each query, the number of residues, number of structural templates (Ntmp), and results from each of the 7 alignment methods, are shown. For each alignment method, the NER4 score (NER), number of gaps in the core alignment (Gp), and number of aligned residues below three RMSD cutoffs (N2,N4,N6) are reported. Each entry in the table represents an average over the number of templates specified in column 3. The averages on the last row refer to the total set of 3,102 structures. In the case of the number of templates, the sum, rather than the average, is given. [file 1471-2105-6-221-S1.PDF]

Query			Default GASH					Meta GASH					DaliLite					CE					Global ASH					No Crossover GASH					High Crossover GASH				
ID	Nres	Ntmp	NER	Gp	N2	N4	N6	NER	Gp	N2	N4	N6	NER	Gp	N2	N4	N6	NER	Gp	N2	N4	N6	NER	Gp	N2	N4	N6	NER	Gp	N2	N4	N6	NER	Gp	N2	N4	N6
1ab8A	208	38	53	46	59	79	84	56	50	64	82	86	51	47	54	80	85	49	38	49	79	84	44	43	46	72	80	49	43	52	78	84	54	53	61	80	85
1bxrA	1104	124	86	140	92	148	170	88	125	96	148	169	73	110	72	130	149	73	63	69	132	154	70	84	74	122	143	83	131	87	144	168	86	142	92	148	171
1frvA	293	97	75	121	75	127	143	76	126	78	128	143	73	121	67	127	143	62	80	50	115	136	68	108	66	117	135	71	117	68	125	142	75	119	75	127	143
1mniA	184	65	104	19	118	129	131	104	15	118	129	131	103	15	115	129	130	101	13	111	128	130	103	18	116	128	130	103	14	116	128	131	104	19	118	129	131
1qgtB	174	235	44	30	51	69	78	46	36	54	71	78	38	32	38	66	77	31	17	24	60	77	35	20	39	58	68	40	29	42	67	77	45	35	51	70	78
1u02A	253	32	71	149	75	121	132	73	156	80	122	130	69	157	69	119	126	49	75	40	96	116	61	122	62	108	121	68	145	70	119	131	71	155	75	122	133
1bgw	709	530	56	108	56	100	118	60	107	63	102	118	52	85	51	92	105	44	51	34	86	106	42	63	43	73	88	52	97	50	96	116	56	111	57	100	118
1dwuA	244	16	74	97	81	110	117	76	87	84	110	116	66	89	68	101	107	62	52	58	98	110	64	49	69	95	108	70	70	74	106	116	74	97	81	111	118
1gqeA	385	495	43	60	46	71	83	45	58	50	73	85	32	29	31	57	68	30	20	22	58	75	34	25	36	56	67	39	54	40	68	82	43	60	47	72	84
1nvbB	422	308	72	101	74	126	144	75	102	80	127	143	68	84	68	119	136	62	71	53	116	135	61	73	60	110	129	68	97	66	124	143	72	102	74	126	144
1r6fA	303	25	49	88	55	78	87	53	94	62	79	85	45	91	47	76	83	26	12	18	51	70	41	60	46	67	76	45	82	49	75	85	49	88	56	78	87
1udyA	416	23	68	32	79	99	105	71	40	82	103	109	60	33	63	95	104	58	24	54	97	108	57	32	65	86	95	64	31	70	98	105	69	39	80	101	108
1bwwA	140	485	85	16	97	101	102	85	16	97	101	101	84	15	96	101	101	83	15	94	101	102	84	15	95	101	101	84	16	96	101	102	85	16	97	101	101
1e03L	454	13	277	24	328	341	343	277	24	328	341	343	276	24	328	341	343	274	24	326	341	343	277	24	327	341	343	271	24	324	341	343	277	24	328	341	343
1kyqB	298	320	56	84	59	95	108	57	79	62	97	108	50	63	50	88	100	45	52	38	86	102	44	56	46	76	89	53	76	54	93	106	56	83	60	96	108
1obaA	369	56	91	113	92	161	179	93	113	96	163	180	85	107	80	155	173	82	86	72	151	166	81	108	76	150	172	87	113	83	159	178	91	113	92	161	179
1sftB	411	240	86	108	85	151	168	88	109	88	153	168	82	99	76	147	163	74	77	63	140	156	73	90	69	134	155	82	102	78	148	166	86	109	85	151	168
**Ave.**	**374**	**3102**	**66**	**75**	**70**	**104**	**116**	**68**	**74**	**74**	**105**	**116**	**60**	**60**	**62**	**98**	**108**	**55**	**42**	**51**	**94**	**109**	**56**	**50**	**59**	**89**	**101**	**62**	**69**	**64**	**102**	**115**	**66**	**76**	**70**	**104**	**116**
