# Supplementary material for: GASH: An improved algorithm for maximizing the number of equivalent residues between two protein structures
Source: BMC Bioinformatics. 2005 Sep 8;6:221. doi: 10.1186/1471-2105-6-221 (PMC1239909; doi:10.1186/1471-2105-6-221)
Supplement: Additional File 2 — Summary of Fischer-Eisenberg set results. For each structure pair, the PDB ID and number of residues, as well as results from each of the 7 alignment methods, are shown. For each alignment method, the NER4 score (NER), number of gaps in the core alignment (Gp), and number of aligned residues below three RMSD cutoffs (N2,N4,N6) are reported. In the last row, the averages over all ten structure pairs are given. [file 1471-2105-6-221-S2.PDF]

**Query-Template Pair**		**Default GASH**					**Meta GASH**					**DaliLite**					**CE**					**Global ASH**					**No Crossover GASH**					**High Crossover GASH**				
**ID(Nres)**	**ID(Nres)**	**NER**	**Gp**	**N2**	**N4**	**N6**	**NER**	**Gp**	**N2**	**N4**	**N6**	**NER**	**Gp**	**N2**	**N4**	**N6**	**NER**	**Gp**	**N2**	**N4**	**N6**	**NER**	**Gp**	**N2**	**N4**	**N6**	**NER**	**Gp**	**N2**	**N4**	**N6**	**NER**	**Gp**	**N2**	**N4**	**N6**
2sim(387)	1nsbA(408)	190	133	205	304	306	190	133	207	305	307	188	135	199	304	306	186	145	185	300	300	189	133	206	304	306	190	137	207	303	305	190	133	207	304	306
1crl(541)	1ede(315)	135	225	152	215	236	135	225	152	215	236	134	225	152	215	236	122	226	116	213	234	129	235	136	220	239	131	219	144	217	238	135	225	152	215	236
1tie(174)	4fgf(133)	84	44	100	116	116	84	44	100	116	116	81	42	97	117	117	82	42	97	117	117	83	44	100	116	116	84	44	100	116	116	84	44	100	116	116
1ten(93)	3hhrB(206)	74	22	86	86	86	74	22	86	86	86	73	19	87	87	87	74	22	86	86	86	74	22	86	86	86	74	22	86	86	86	74	22	86	86	86
1cld(182)	2rhe(126)	72	16	87	102	102	71	15	86	102	103	70	17	85	102	102	68	17	81	102	102	69	15	83	102	103	70	14	84	102	103	72	16	87	102	102
1bgeB(167)	2gmfA(128)	67	41	76	106	116	68	42	79	106	116	65	41	72	107	116	56	38	50	103	117	61	34	67	99	109	64	40	74	105	117	67	42	79	106	116
1cewI(114)	1molA(97)	64	32	78	83	83	65	37	79	80	80	64	36	79	81	81	62	37	77	80	80	64	32	78	83	83	64	32	78	83	83	64	32	78	83	83
2aza(151)	1paz(130)	63	49	77	87	87	64	49	78	87	87	62	48	76	87	87	58	47	67	88	88	62	49	76	87	87	61	47	74	88	88	63	49	77	87	87
3hlaB(107)	2rhe(126)	54	35	62	84	85	54	35	63	84	85	53	35	59	84	85	49	36	44	85	86	53	34	62	84	85	50	35	49	84	85	54	35	62	84	85
1fxiA(99)	1ubq	44	38	52	65	67	45	37	52	66	67	44	37	51	66	67	42	37	43	67	67	44	38	51	65	67	44	38	51	65	67	44	38	52	65	67
**Average**		**84**	**63**	**97**	**124**	**128**	**85**	**63**	**98**	**124**	**128**	**83**	**63**	**95**	**125**	**128**	**79**	**64**	**84**	**124**	**127**	**82**	**63**	**94**	**124**	**128**	**83**	**62**	**94**	**124**	**128**	**84**	**63**	**98**	**124**	**128**
